# Efficacy of pegylated interferon α2a in patients without HBeAg loss after the withdrawal of long-term lamivudine therapy

**DOI:** 10.1186/1743-422X-10-21

**Published:** 2013-01-15

**Authors:** Xu-Qing Zhang, Hui-Yan Zhang, Jian-Ping You, Qing Mao

**Affiliations:** 1 Department of Infectious Diseases, Southwest Hospital, Third Military Medical University, Chongqing 400038, P.R. China

**Keywords:** Chronic hepatitis B, Pegylated interferon α2a, Lamivudine, Biochemical breakthrough

## Abstract

**Background:**

Improving the HBe seroconversion rate of patients without HBeAg loss after long-term lamivudine therapy has become an urgent clinical problem that we have to face. Unfortunately, there is no consensus on the mananement of these patients. The aim of this study was to evaluate the efficacy of pegylated interferon (PEG-IFN) α2a in patients without HBeAg loss after the withdrawal of long-term lamivudine therapy.

**Methods:**

Fifty patients with chronic hepatitis B without the loss of HBeAg after ≥96 weeks of lamivudine treatment were enrolled to withdraw from treatment to induce a biochemical breakthrough. Patients who achieved a biochemical breakthrough within 24 weeks received 48-weeks of PEG-IFN α2a therapy, and were then assessed during a subsequent 24-week follow-up period.

**Results:**

Forty-three (86.0%) patients achieved a biochemical breakthrough within 24 weeks of lamivudine withdrawal. The rates of combined response (both undetectable HBV DNA and HBeAg loss) and HBsAg loss were alone 51.2% and 20.9%, respectively after 48 weeks of PEG-IFN α2a therapy, and 44.2% and 18.6%, respectively, at 24 weeks after treatment cessation. The end-of-treatment combined response rate was 65.4% among patients with a baseline HBsAg <20,000 IU/mL, which was significantly higher than 29.4% of patients with HBsAg ≥20,000 IU/mL (P=0.031). For patients with HBsAg levels <1,500 IU/mL at 12 and 24 weeks therapy, the end-of-treatment combined response rate was 68.2% and 69.0%, which were both significantly higher than patients with HBsAg ≥1,500 IU/mL (33.3% and 14.3%; P=0.048 and 0.001). The end-of-treatment combined response rate was significantly higher among patients with HBV DNA<10^5^ copies/mL (76.2%) compared to patients with HBV DNA ≥10^5^ copies/mL (27.3%) after 24 weeks of therapy (P=0.004).

**Conclusion:**

Retreatment with PEG-IFN α2a was effective and safe for patients without HBeAg loss after the withdrawal of long-term lamivudine therapy. HBsAg levels at the baseline, 12 and 24 weeks of therapy, and HBV DNA levels at 24 weeks of therapy, can predict the effect of PEG-IFN α2a after 48 weeks of therapy.

## Background

Chronic infection with hepatitis B virus (HBV) is a major global health problem, which affects more than 400 million people worldwide [1,2]. Approximately 15–40% of infected patients develop cirrhosis, liver failure or hepatocellular carcinoma (HCC). Appropriate antiviral treatment has been confirmed to be effective in preventing advanced liver disease and reducing the number of HBV-related deaths.

The current treatment options for chronic HBV infection consist of interferon-α (IFN-α) and nucleos(t)ide analogues (NUCs), such as lamivudine, adefovir, entecavir and telbivudine. Since lamivudine was initially approved for the treatment of chronic HBV infection in China, several million affected patients have received long-term antiviral treatment with NUCs to prevent disease progression. Most patients hope that the period of antiviral treatment is temporary. In HBeAg- positive patients, durable HBe seroconversion is a satisfactory end-point as it is associated with an improved prognosis [1]. However, it is very difficult for most HBeAg positive patients to meet the criteria for the cessation of lamivudine treatment. In these patients, the rate of HBe seroconversion with lamivudine treatment has been reported to be 16-17% at 48 weeks, 17-29% at 96 weeks, 23-40% at 144 weeks, and 28-47% at 192 weeks [1-4]. Unfortunately, extended treatment with lamivudine is associated with the development of drug resistance. The cumulative incidence of HBV resistance to lamivudine is 24% at 1 year, 38% at 2 years, 49% at 3 years, 67% at 4 years, and 70% at 5 years. Therefore, it is very important to institute new treatment strategies to improve the rate of HBeAg loss or HBe seroconversion for patients without HBeAg loss after long-term lamivudine treatment. However, there are no definitive guidelines regarding whether lamivudine therapy should be continued or a new treatment should be commenced and the optimal stopping point for receiving lamivudine treatment has yet to be determined.

IFN-α exerts an antiviral effect by degrading viral mRNA and proteins, and upregulates the immunological response to HBV by enhancing human leukocyte antigen class I expression on hepatocytes, its use has been confirmed as effective in the treatment of chronic HBV infection [1,5-8]. The use of pegylated interferon (PEG-IFN) α has certain advantages over NUCs, which include the absence of resistance, higher rates of HBeAg loss and seroconversion, and higher rates of both sustained off-treatment virological responses and HBsAg loss [1]. For HBeAg-positive patients with PEG-IFNα, higher rates of HBeAg loss or seroconversion are associated with higher pre-treatment serum alanine aminotransferase (ALT) levels. Additionally, post-treatment ALT flares with viral proliferation often occur following the withdrawal of lamivudine. Thus, a new retreatment strategy that involves switching from lamivudine during a withdrawal-induced ALT flare to PEG-IFN α2a therapy is a reasonable protocol for patients without HBeAg loss after long-term lamivudine treatment. However, neither the efficacy nor safety of this new treatment strategy has been fully demonstrated. The aim of our study was to evaluate the use of PEG-IFN α2a therapy for the retreatment of patients with chronic HBV infection after a lamivudine withdrawal-induced biochemical breakthrough. These patients were without HBeAg loss after more than 96 weeks lamivudine treatment.

## Methods

### Selections of patients and study design

Inclusion criteria included: i) age 18–60 years; ii) continuous lamivudine treatment >96 weeks; iii) serum HBV DNA levels <10^3^ copies/mL;iv) positive for HBsAg and HBeAg, but negative for anti-HBs and anti-HBe;v) normal liver function tests;vi) no history of liver failure or cirrhosis,and the lack of cirrhosis confirmed by a computed tomography scan; vii) neutrophil count ≥ 1.5×10^9^/L and platelet count ≥100×10^9^/L. The exclusion criteria included: i) superinfection with hepatitis A, C, D or E, cytomegalovirus, HIV, or Epstein–Barr virus as confirmed by enzyme-linked immunosorbent assay; ii) other liver diseases such as alcoholic liver disease, drug-induced hepatitis, Wilson disease and autoimmune hepatitis; iii) severe medical or psychiatric illness; iv) a history of diabetes, cardiac disease, hypertension, or renal, pulmonary disease or thyroid disease; v) pregnant or breastfeeding women; vi) an unwillingness or inability to provide informed consent or fulfill the the requirements of the study.

**Figure 1:**
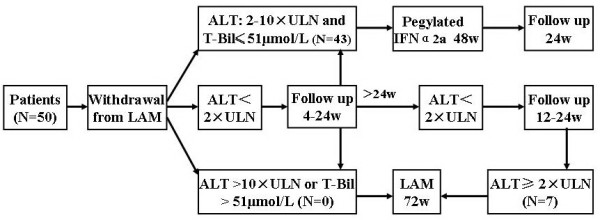
**Study design.** Liver function was tested 4-weekly within 24 weeks of lamivudine withdrawal and 12-weekly after 24 weeks of lamivudine withdrawal. ALT: alanine aminotransferase; ULN: upper limit of normal; IFN: interferon; T-Bil: total bilirubin; LAM: lamivudine.

Fifty outpatients with chronic hepatitis B (35 male, 15 female; median age, 33 years; range, 20–52 years) were recruited at the Southwest Hospital, Third Military Medical University, China, between January 2006 and June 2010. They were asked to withdraw from lamivudine and any other medications. Liver function tests were performed every 4 weeks. When a biochemical breakthrough was observed, patients underwent a serum HBV DNA test and retreatment with either PEG-IFN α2a (180 μg, once per week) or lamivudine, in accordance with the study design shown in Figure 1. This study was conducted in accordance with the guidelines of the Declaration of Helsinki. The ethics committee of Southwest Hospital approved the study protocol, and all patients gave witnessed, written informed consent.

### Laboratory assays

Serum HBsAg and anti-HBs, HBeAg and anti-HBe were tested using the ARCHITECT (Abbott Laboratories). The range of HBsAg detection was 0.05 to 250 IU/mL. Serum HBV DNA was tested using real-time PCR (Amplicor HBV Monitor Test, Roche Diagnostics, Mannheim, Germany; the range of HBV DNA detection was 10^3^ to 10^9^ copies/mL). We performed HBV genotyping by direct PCR sequencing in a residual serum sample taken from each patient at the baseline timepoint before they started the PEG-IFN α2a treatment.

### Evaluation of therapeutic effects and safety

We defined virological response as HBV DNA <10^3^ copies/mL, serological response as HBeAg loss, biochemical response as normalization of ALT level, and a combined response as HBV DNA < 10^3^ copies/mL and HBeAg loss. We assessed both end-of-treatment combined responses after 48 weeks of PEG-IFN α2a therapy and sustained combined responses at 24 weeks after the end of treatment. The primary end point was sustained combined responses. Secondary end points were end-of-treatment combined responses and HBsAg loss.

In order to evaluate the safety of this study, serum levels of ALT and total bilirubin (T-Bil) were tested 4-weekly during lamivudine withdrawal and the first 12-weeks of PEG-IFN α2a therapy. Patients undergoing PEG-IFN α2a therapy were then tested every 12 weeks during a further 60-week period. Peripheral leukocytes and platelets were tested at weeks 4, 8, 12, 24, 48 and 72 of therapy. Serum free thyroxin 3 and 4 (FT3 and FT4), thyroid stimulating hormone (TSH) were tested at the baseline, then 12 weekly until the end of treatment. The abnormal laboratory tests results and symptomatic adverse effects were recorded and closely monitored at each follow-up visit. We assessed all adverse events for the likelihood of causality by the study drug.

### Statistical analysis

We performed all statistical tests with SPSS, version 10.0 (SPSS, Inc., Chicago, IL, USA). We expressed continuous variables as the median±standard deviation. The levels of serum HBV DNA and HBsAg were logarithmically transformed for the analyses. A *P-*value less than 0.05 was considered statistically significant. All statistical tests were two-sided.

## Results

### Biochemical breakthrough induced by lamivudine withdrawal

**Figure 2:**
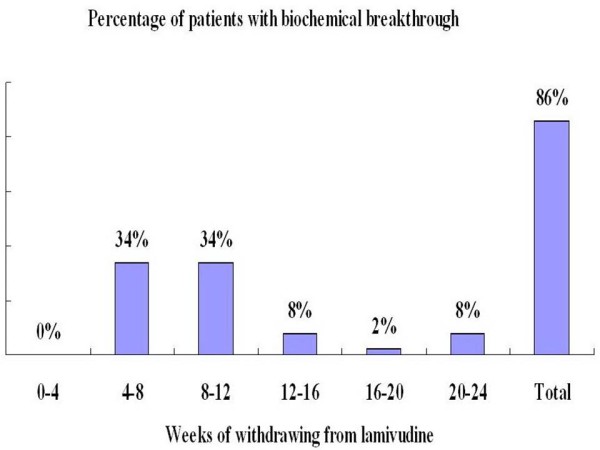
Percentage of patients with biochemical breakthrough occurred at the different time after lamivudine withdrawal.

Forty-three (86.0%) patients were observed to have a biochemical breakthrough (ALT levels ≥2×ULN) within 24 weeks of lamivudine withdrawal, and to have a virological breakthrough (HBV DNA ≥10^5^ copies/mL). They received a treatment with PEG-IFN α2a (Figure 2). Another seven patients received a retreatment with lamivudine; a subsequent biochemical breakthrough was observed in four patients (8.0%) after 36 weeks of the initial lamivudine withdrawal, and in three patients (6.0%) after 48 weeks of lamivudine withdrawal. The characteristics of the patients in this study are shown in Table 1. With the exception of the median ALT levels at the time of the observed biochemical breakthrough observed, there were no statistically significant difference with regards to age, gender, genotype B, HBV DNA levels, and the duration of prior lamivudine therapy between patients who had a biochemical breakthrough occurred within 24 weeks of lamivudine withdrawal and those in which it occurred after 24 weeks.

**Table 1:** The characteristics of the studied patients at the occurrence of biochemical breakthrough

**Characteristics**	**Weeks of biochemical breakthrough**		
	**≤24 (n=43)**	**>24 (n=7)**	**p value**
Age, yrs	32.5±7.9	37.1±5.8	NS
Men, n(%)	30(69.8%)	6(85.7%)	NS
Duration of previous LAM	126±43	134±37	NS
treatment (weeks)	(104–312)	(104–208)	
ALT level, n(%)			
2-3×ULN	21(48.8%)	1(14.3%)	NS
≥3×ULN	22(51.2%)	6(85.7%)	
ALT level,IU/L	190±126	318±172	0.024
HBV DNA ≥1×10^7^ copies/mL,%	29(67.4%)	6(85.7%)	NS
HBV DNA(log_10_ copies/mL)	7.3±0.8	7.7±0.9	NS
HBV genotype, n (%)			
B	30(69.8%)	5(71.4%)	NS
C	12(27.9%)	2(28.6%)	
B and C	1( 2.3%)	0(0)	

ALT: alanine aminotransferase; ULN: upper limit of normal; LAM: lamivudine;

Weeks of biochemical breakthrough: Interval from lamivudine withdrawal to biochemical breakthrough; NS: no significant difference.

### Therapeutic efficacy of PEG-IFN α2a

**Figure 3:**
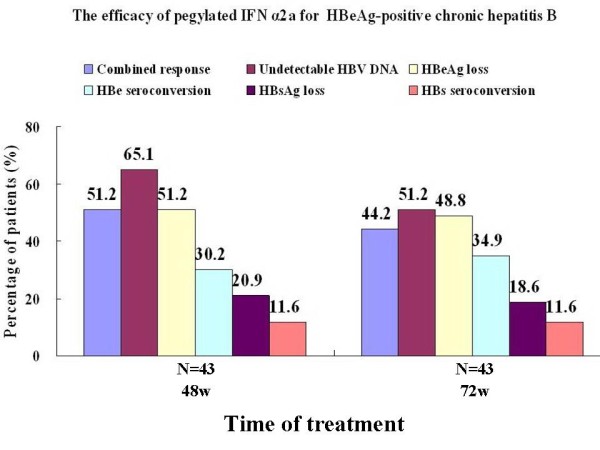
The efficacy of pegylated interferon α2a for patients with HBeAg positive chronic hepatitis B.

Among 43 patients treated with PEG-IFN α2a therapy, the rates of combined response (both HBV DNA <10^3^ copies/ml and HBeAg loss) and HBsAg loss were 51.2% and 20.9%, respectively, at the end of 48 weeks therapy, and 44.2% and 18.6%, respectively, 24 weeks after the end of treatment. The rates of undetectable HBV DNA, HBeAg seroconversion and loss, and HBsAg seroconversion were shown in Figure 3. Among six patients who only showed a virological response at the end of 48 weeks therapy, three patients had a sustained virological response by 24 weeks after the end of treatment, but no patient showed HBeAg loss. Among the 15 patients without a virological response or HBeAg loss at the end of 48 weeks therapy, only one patient had HBeAg loss and seroconversion 24 weeks after the end of treatment, but his HBV DNA level was 3.108×10^3^ copies/mL. Among the 22 patients with a combined response at the end of 48 weeks therapy, 19 (86.4%) patients had a sustained combined response 24 weeks after the end of treatment. A normalization of the ALT levels was observed in 25 patients (58.1%) at the end of 48 weeks treatment, and among 32 patients (74.4%) by 24 weeks after the end of treatment.

All seven patients who received a retreatment with lamivudine had an undetectable HBV DNA at 48 weeks and 72 weeks of treatment, but none showed HBeAg loss, HBe seroconversion, or HBsAg loss during the 72 weeks of lamivudine treatment.

### The combined response and baseline characteristics of patients

**Figure 4:**
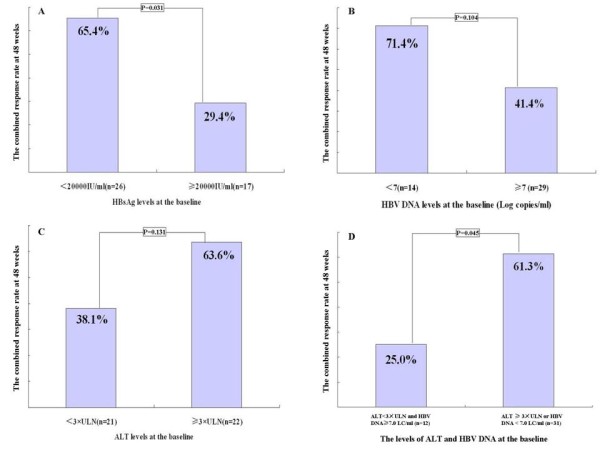
**The association of the combined response rate at the end of 48 weeks pegylated interferon α2a treatment with serum levels of ALT, HBV DNA and HBsAg at the baseline.** ALT: alanine aminotransferase; ULN: upper limit of normal; LC/mL: Log_10_ copies/ml.

The baseline characteristics of patients with or without combined response at the end of 48 weeks PEG-IFN α2a therapy are shown in Table 2. There was no statistically significant difference in the median levels of serum HBsAg between patients with or without a combined response at the end of the 48-week treatment (P=0.070, Table 2), but the rate of combined response was significantly higher among patients with HBsAg <20,000 IU/mL than in patients with HBsAg levels ≥20,000 IU/mL (P=0.031, Figure 4A). The rate of combined response was higher among patients with HBV DNA levels <10^7^ copies/mL than in patients with HBV DNA levels ≥10^7^ copies/mL, but this was not significantly different (P=0.104, Figure 4B). The rate of combined response was also higher among patients with ALT levels ≥3×ULN compared to patients with ALT levels <3×ULN, but again this difference was not significant (P=0.131, Figure 4C). However, the rate of combined response was significantly higher among patients with ALT levels ≥3×ULN or HBV DNA levels <10^7^ copies/mL than in patients with both ALT levels <3×ULN and HBV DNA levels ≥10^7^ copies/mL (P=0.045, Figure 4D). There were no significantly differences with age, gender and genotype B between patients that had a combined response and those that did not (Table 2).

**Table 2:** Baseline characteristics of patients with pegylated interferon α2a

**Characteristics**	**Combined responses at 48 weeks**		
	**Yes (n=22)**	**No (n=21)**	**p value**
Age, yrs	32.7±8.7	32.2±7.1	NS
Men, n(%)	13(59.1%)	17(81.0%)	NS
ALT level, n(%)			
≥3×ULN	14(63.6%)	8(38.1%)	NS
ALT level,IU/L	209±137	171±113	NS
HBV DNA ≥1×10^7^ copies/mL,%	12(54.5%)	17(81.0%)	NS
HBV DNA(log_10_ copies/mL)	7.1±0.7	7.5±1.0	NS
HBsAg ≥2×10^4^ IU/ml,%	5(22.7%)	12(57.1%)	0.031
HBsAg(log_10_ IU/mL)	3.8±0.8	4.2±0.6	0.070
HBV genotype, n (%)			
B	15(68.2%)	15(71.4%)	NS
C	7(31.8%)	5(23.8%)	
B and C	0(0)	1( 4.8%)	

ALT: alanine aminotransferase; ULN: upper limit of normal; combined responses at 48 weeks: both HBV DNA< 1000 copies/ml and HBeAg loss at the end of 48 weeks treatment; NS: no significant difference.

### The predictive values of HBV DNA and HBsAg levels during treatment

**Figure 5:**
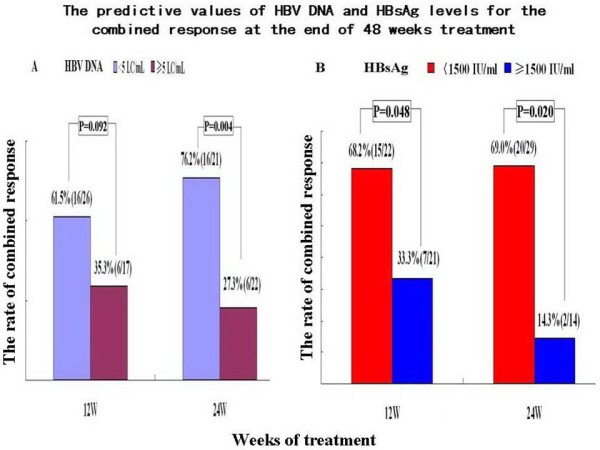
**The predictive values of HBV DNA and HBsAg levels during treatment for the combined response (HBeAg loss and undetectable HBV DNA) at the end of 48 weeks treatment.** LC/ml: Log_10_ copies/ml.

The rate of combined response at the end of 48 weeks treatment was 61.5% among 26 patients with HBV DNA <10^5^copies/mL and 35.3% among 17 patients with HBV DNA ≥10^5^copies/mLat 12 weeks of therapy, but this was not a significant finding (P=0.092). For patients with HBV DNA <10^5^ at 24 weeks of therapy, the rate of combined response at the end of 48 weeks of therapy reached 76.2%, which was significantly higher than in patients (27.3%) with HBV DNA ≥10^5^copies/mL, P=0.004, shown in Figure 5A. The baseline mean serum HBV DNA was 7.20 ± 0.94 and 7.08±0.91 LC/mL among patients with HBV DNA <10^5^copies/mL at 12 weeks and 24 weeks of therapy, which were lower than among patients with HBV DNA ≥10^5^copies/mL (7.53 ± 0.76 and 7.56±0.79 LC/mL) at 12 weeks and 24 weeks of therapy; these were not significant differences at either time point (P=0.232, 0.072). For patients with HBsAg <1,500 IU/mL at 12 weeks and 24 weeks of therapy, the rates of combined response at the end of 48 weeks were 68.2% and 69.0%, which were significantly higher than in patients with HBsAg ≥1,500 IU/mL (33.3% and 14.3%; P=0.048, 0.001; Figure 5B). The baseline mean serum HBsAg was 3.63 ± 0.82 and 3.80 ± 0.79 log_10_ IU/mL in patients with HBsAg <1,500 IU/mL at 12 weeks and 24 weeks of therapy, which was significantly lower than among patients with HBsAg ≥1,500 IU/mL (4.43 ± 0.43 and 4.48 ± 0.44 Log_10_ IU/mL; P=0.0003, 0.004).

The rates of HBsAg loss at the end of 48 weeks treatment were 31.0% (9/29), 38.9% (7/18) and 63.6% (7/11) among patients with HBsAg < 1500, 100,10 IU/mL, but only reached 0 (0/14), 8.0% (2/25) and 6.3% (2/32) among patients with HBsAg ≥1500, 100,10 IU/mL at 24 weeks of therapy, having statistically significant difference (P=0.020, 0.038, 0.0003).

The rate of sustained combined response was significantly higher among patients with HBV DNA <10^3^ copies/mL (67.9%, 19/28) or HBsAg <1500 IU/mL (59.4%, 19/32) compared to that of patients with HBV DNA ≥10^3^ copies/mL (0, 0/15) or HBsAg ≥1500 IU/mL (0, 0/11) at the end of 48 weeks treatment, P<0.01.

### Safety

From the withdrawal of lamivudine to the initiation of PEG-IFN α2a treatment, none of 50 patients had any evidence of hepatic decompensation or a severe acute exacerbation of chronic HBV infection characterized by ALT levels being >10×ULN or total bilirubin levels being >51 μmol/L. During 48 weeks treatment with PEG-IFN α2a, four (9.3%) patients developed a severe ALT flare-up (ALT levels >10×ULN), of which two patients developed it at 4 weeks of treatment (749 IU/L and 535 IU/L, respectively), one patient developed it at 8 weeks of treatment (552 IU/L), and another patient at 12 weeks of treatment (616 IU/L). At 24 weeks after the end of treatment, two (4.7%) patients developed a severe ALT flare-up (487 and 2042 IU/L). However, during 48 weeks treatment with PEG-IFN α2a and 24-week follow up period, T-Bil levels did not exceed 51 μmol/L among 43 patients. Other adverse symptoms and events related to the use of PEG-IFN α2a included upper respiratory tract symptoms (55.8%, 24/43), fever (69.8%, 30/43), alopecia (32.6%, 14/43), abdominal discomfort (34.9%, 15/43), malaise (69.8%, 30/43), headache (65.1%, 28/43), myalgia (53.5%, 23/43), arthralgia (27.9%, 12/43), reduced appetite (25.6%, 11/43), local erythematous reactions (9.3%, 4/43), weight loss (14.0%, 6/43), a decreased neutrophil count (46.5%, 20/43) and a decreased platelet count (51.2%, 22/43). No patient required a dosage adjustment for PEG-IFN α2a, and no patient developed thyrotoxicosis or hypothyroidism.

## Discussion

Improving the HBe seroconversion rate of patients without HBeAg loss after long-term lamivudine therapy has become an urgent clinical problem that we have to face. Unfortunately, there is no consensus on the management of these patients. In this study, we administered a new retreatment strategy by switching patients without HBeAg loss after long-term lamivudine therapy with a withdrawal-induced ALT flare to PEG-IFN α2a therapy. The rationale of this new retreatment strategy was that lamivudine withdrawal usually induces a biochemical breakthrough and a host antiviral immune response, and the biochemical breakthrough characterized by high serum ALT levels is associated with a better response to IFN-α [1,9-13]. Our results also showed that lamivudine withdrawal could induce biochemical breakthroughs, and 86% patients relapsed within 24 weeks after the discontinuation of the initial lamivudine therapy. These findings were similar to those reported by Shin JW, et al. [11], and indicated that lamivudine withdrawal provided a new opportunity for treatment with IFN-α in patients without HBeAg loss.

Although patients who relapse after lamivudine withdrawal can be treated with any available effective therapeutic agent, but treatment with NUCs has an indefinite duration and causes low rates of HBeAg seroconversion or loss, and furthermore, HBeAg seroconversion induced by lamivudine retreatment is not permanent [1,10,11]. Therefore, retreatment with lamivudine or other NUCs is not a good option for patients who relapse after lamivudine withdrawal. However, retreatment with standard IFNα was reported to have a similar efficacy to the first IFN-α treatment in HBeAg-positive chronic hepatitis B, and the efficacy of PEG-IFN α2a is better than standard IFNα in treating HBeAg-positive chronic hepatitis B [14]. Consequently, PEG-IFN α2a should be considered as the first choice for patients who relapse after lamivudine withdrawal. Wang Z et al. reported that the rates of HBeAg loss and seroconversion were 18.9% and 13.2% after 1 year of retreatment with a combination of lamivudine plus adefovir (36.0% and 28%) or lamivudine alone (3.6% and 0%) for patients who relapsed after lamivudine withdrawal [15]. However, our results showed that the rates of combined response, HBeAg loss and seroconversion reached 51.2%, 51.2% and 30.2%, respectively, at the end of 48 weeks therapy with PEG-IFN α2a in patients who relapsed after lamivudine withdrawal, and none achieved HBeAg seroconversion and loss among a further seven patients whoreceived retreatment with 72 weeks lamivudine. These finding indicated that the effect of PEG-IFN α2a was better than retreatment with NUCs for patients who relapsed after lamivudine withdrawal. More importantly, HBeAg loss and seroconversion induced by lamivudine treatment was not permanent, as 40%-60.7% patients who had achieved HBeAg seroconversion or loss with lamivudine relapsed after lamivudine withdrawal [11,16]. Our current results showed that the rates of combined response, HBeAg loss and seroconversion were 44.2%, 48.8% and 34.9% at 24 weeks after the end of 48 weeks PEG-IFN α2a treatment, and 86.4% patients who had a combined response at the end of 48 weeks therapy had a sustained combined response at 24 weeks after the end of treatment. This indicated that the HBeAg loss and seroconversion induced by PEG-IFN α2a treatment was more durable than that induced by lamivudine for patients who relapsed after lamivudine withdrawal. Otherwise, the rate of HBsAg loss reached 20.9% at the end of 48 weeks therapy with PEG-IFN α2a, and 18.6% at 24 weeks after the end of treatment in this study, which was higher than the 0-3% reported with NUCs treatment [1,11,15]. Therefore, our results preliminarily confirmed that the retreatment strategy of switching from a lamivudine withdrawal-induced ALT flare to PEG-IFN α2a therapy provided a new opportunity to obtain a sustained combined response off-treatment and a chance of HBsAg loss in patients who had not achieved this after long-term lamivudine treatment.

Recent studies indicated that HBsAg levels at the baseline and during treatment can predict the effect of PEG-IFN α in the treatment of chronic hepatitis B [16,17]. Our results also showed that the rate of combined response at the end of 48 weeks therapy with PEG-IFN α2a was significantly higher among patients with baseline levels of HBsAg <20,000 IU/mL than patients with baseline levels of HBsAg ≥20,000 IU/mL, and was significantly higher among patients with HBsAg <1,500 IU/mL at 12 weeks and 24 weeks of therapy than patients with HBsAg ≥1,500 IU/mL. The lower HBsAg levels after 24 weeks of therapy, the higher rate of HBsAg loss at the end of 48 weeks of therapy. These results further confirmed that HBsAg levels at the baseline and during treatment could predict the rate of a combined response at the end of 48 weeks therapy. Furthermore, HBsAg levels after 24 weeks of therapy may be helpful in predicting HBsAg loss at the end of 48 weeks. Low viral load and high serum ALT levels at the baseline have been reported to be associated with a better response to IFNαin other studies [1,6,7]. However, a statistically significant difference was not observed in the rate of combined response at the end of 48 weeks therapy with PEG-IFN α2a between patients with baseline HBV DNA ≥10^7^ copies/mL and baseline HBV DNA <10^7^ copies/mL. No significant difference was also found between patients with baseline ALT ≥3×ULN and those with baseline ALT <3×ULN in this study, which indicated that baseline ALT level alone or HBV DNA level alone was not a good indicators for predicting the effect of PEG-IFN α2a therapy. Possible reasons for these results included the fact that the sample size in this study was too small to achieve a statistically significant difference, and that the interaction of both ALT and HBV DNA affected the response to treatment. Our results showed that the rate of combined response was significantly higher among patients with baseline ALT ≥3×ULN or HBV DNA <10^7^copies/mL than patients with both baseline ALT <3×ULN and HBV DNA ≥10^7^ copies/mL, which indicated that combined baseline ALT and HBV DNA levels may predict the effect of PEG-IFN α2a therapy, and was better than ALT or HBV DNA levels alone. Our results also showed that the rate of combined response at the end of 48 weeks of therapy was significantly higher among patients with HBV DNA <10^5^ at 24 weeks of therapy than those with levels ≥10^5^copies/mL, indicated that the HBV DNA level at 24 weeks of treatment might be able to predict the response rate at the end of 48 weeks of PEG-IFN α2a therapy, this had been described in other studies [1,13]. Our results did not showed any significant differences with regards to age, gender and genotype B among patients with or without a combined response. However, younger female patients with genotype B have been shown to be more likely to have a good response to interferon by other studies [1].

In this study, we also found that the rate of a sustained combined response at 24 weeks after the end of treatment was closely associated with HBeAg status, HBV DNA levels and HBsAg levels at the end of 48 weeks therapy. Therefore, we suggested that extended treatment with PEG-IFN α2a may be helpful for patients who only achieved virological response or combined response but having high HBsAg levels at the end of 48 weeks therapy, in order to achieve a better sustained combined response. Long-term maintained therapy with NUCs should be considered for patients without virological response or HBeAg loss at the end of 48 weeks therapy.

Hepatic decompensation and severe acute exacerbation of chronic hepatitis B induced by lamivudine withdrawal are major clinical concerns. In this study, none of 50 patients had a severe acute exacerbation of chronic hepatitis B during the 48 weeks follow-up after lamivudine withdrawal, which indicated that the withdrawal of lamivudine was safe for these patients without HBeAg loss and underlying liver cirrhosis, if liver function tests are monitored monthly for 24 weeks after lamivudine withdrawal. Our results also indicated that lamivudine withdrawal-induced hepatic decompensation and severe acute exacerbation were uncommon events and could be controlled for patients without underlying liver cirrhosis, which was in accordance with other previous reports [9]. This study showed that interferon-α can be given safely and effectively in patients with ALT levels that were 2–10 times ULN and serum total bilirubin ≤51 μmol/L. For patients with ALT levels > 10 × ULN or serum total bilirubin >51 μmol/L, lamivudine or other NUCs was considered to be given [9]. For patients with a severe acute exacerbation, combination therapy with NUCs and corticosteroids have been confirmed to be effective in preventing the disease progression into hepatic decompensation [18,19]. In this study, the incidences of adverse events associated with PEG-IFN α2a treatment were similar to those reported in other studies [6].

In conclusion, a retreatment strategy of switching to PEG-IFN α2a therapy in patients with lamivudine withdrawal-induced ALT flare was shown to be safe and efficacious for patients without HBeAg loss and underlying liver cirrhosis after long-term lamivudine treatment. A sustained combined off-treatment response and HBsAg loss were achieved. HBsAg levels at the baseline, 12 and 24 weeks of therapy, and HBV DNA levels at 24 weeks of therapy were critical factors for predicting the effect of 48 weeks therapy with PEG-IFN α2a. However, our conclusions remain conservative because of the small sample size and nonrandomized controlled study.

## Abbreviations

DNA: Deoxyribonucleic acid; HBV: Hepatitis B Virus; HBsAg: Hepatitis B surface antigen; HBeAg: Hepatitis B e antigen; NUCs: Nucleo(s)tide analogues; PEG-IFN: Pegylated interferon; ALT: Alanine aminotransferase; ULN: Upper limit of normal; T-Bil: Total bilirubin; PCR: Polymerase chain reaction; FT3 and FT4: Free thyroxin 3 and 4; TSH: Thyroid stimulating hormone.

## Competing interests

There are no conflicts of interest to disclose for all authors. There are not any commercial relationships with other departments except Southwest Hospital of Third Military Medical University.

Financial competing interests

1. In the past five years, we did not received any reimbursements, fees, funding, or salary from any organization that may in any way gain or lose financially from the publication of this manuscript. There is not any organization financing this manuscript (including the article-processing charge).

2. We do not hold any stocks or shares in an organization that may in any way gain or lose financially from the publication of this manuscript, either now or in the future.

3. We are currently applying for any patents relating to the content of the manuscript. We did not receive reimbursements, fees, funding, or salary from any organization that holds or has applied for patents relating to the content of the manuscript.

4. We have not any other financial competing interests.

Non-financial competing interests

There are not any non-financial competing interests (political, personal, religious, ideological, academic, intellectual, commercial or any other) to declare in relation to this manuscript.

## Authors’ contributions

XQZ conceived of the study, participated in its design, the selection and treatment of patients, performed the statistical analysis and drafted the manuscript. HYZ collected the clinical data of all patients, JPY worked for follow up of patients. QM conceived of the study, participated in its design and revised this manuscript. All authors read and approved the final manuscript.
